# Fulvestrant-Big Mac Index: Defining Inequality in Oncology

**DOI:** 10.1200/GO.21.00047

**Published:** 2021-05-06

**Authors:** David O'Reilly, Yassmeen Abu Al-Saud, Cian Ronayne, Seamus O'Reilly

**Affiliations:** David O'Reilly, MB BCh, and Yassmeen Abu Al-Saud, MD, Department of Medical Oncology, Cork University Hospital, Wilton, Cork, Ireland; Cian Ronayne, MPharm, MPSI, Department of Pharmacy, Cork University Hospital, Wilton, Cork, Ireland and Seamus O'Reilly, MD, PhD, Department of Medical Oncology, Cork University Hospital, Wilton, Cork, Ireland

## TO THE EDITOR:

Patel et al^1^ eloquently present compelling evidence that 250 mg of fulvestrant may be a sufficient dose in resource-limited settings. Their work highlights the disparity between cost and efficacy of the 250 mg and 500 mg dosing schedules of fulvestrant in India.

Their work provoked our consideration of how disparities in fulvestrant pricing may occur between different countries. The Big Mac Index uses the cost of a Big Mac to estimate the purchasing power parity of a currency.^2^ How many Big Macs could I buy for the cost of 500 mg of fulvestrant? How many 500 mg of fulvestrant doses could I buy with a monthly salary? How does this vary between countries?

We performed an analysis of the cost of fulvestrant, the cost of Big Macs, and the relationship of these costs with monthly salaries in 13 countries. The countries were chosen to represent a mixed economic demographic and continental distribution. The lowest available price of fulvestrant (generic or brand) was included. We contacted medical oncology colleagues in the listed countries to assist with estimating the cost of 500 mg of fulvestrant. There is a lack of transparent sources for pricing in each country, and the authors' felt contacting colleagues was most likely to yield accurate results. However, our inability to access a central resource with pricing is a limitation of this analysis. Monthly salary and Big Mac prices were included in our analysis.^2,3^

**FIG 1:**
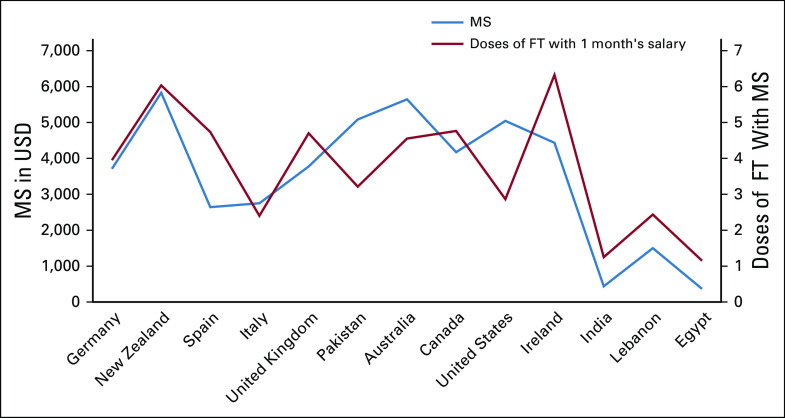
The relationship between the cost of fulvestrant (500 mg) and median MS in 13 countries. FT, fulvestrant; MS, monthly salary; USD, US dollars.

Our findings highlight the wide disparity in fulvestrant costs between different countries. For example, in the United States, the cost of fulvestrant exceeds $1,600 in US dollars, whereas it is < $200 in US dollars for 500 mg in Pakistan (full list of prices available). However, these crude figures do not consider the purchasing power of patients with cancer in these countries. The cost of fulvestrant is more likely to be a higher proportion of monthly income in lower-income countries (Fig 1). For example, in Egypt, the cost of fulvestrant is equivalent to just over 1 month of salary. This is in contrast to our own country (Ireland) where 1 month's salary provides purchasing power for 6 months' supply of fulvestrant. This analysis does not consider that we are also fortunate in many developed countries that our health service sponsors the cost of these medicines.

**FIG 2:**
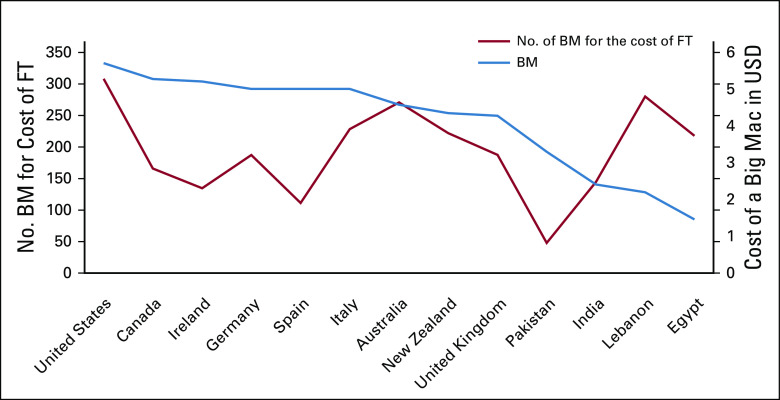
The FT-BM Index. The relationship between the cost of FT and the cost of a BM in 13 countries. BM, Big Mac; FT, fulvestrant; USD, US dollars.

Our novel Fulvestrant-Big Mac index suggests that inequity of pricing in contrast to purchasing power may not be as profound as our initial results would suggest (Fig 2). For example, in Spain, we could buy 111 Big Macs for one dose of fulvestrant (500 mg). However, this increases up to 144 in India.

Patel et al^1^ describe the very significant financial burden synonymous with cancer care. Regardless of the wealth of a country, monies spent on medication have a significant opportunity cost and reduces funds that could be spent on staffing, infrastructure, health promotion, and prevention (eg, vaccination).

Economics is the science of how individuals or organisations with limited resources make decisions. Unfortunately, a diagnosis of cancer is not a choice. We sadly live in a world where the treatment of disease is traded in the same currency as gold, diamonds, and Big Macs. The authors would advocate for a system of pharmaceutical pricing that ensures value for patients and taxpayers regardless of geographical location, economic status, or indeed the price of a Big Mac.
